# Stem cell therapy and tissue engineering for correction of congenital heart disease

**DOI:** 10.3389/fcell.2015.00039

**Published:** 2015-06-30

**Authors:** Elisa Avolio, Massimo Caputo, Paolo Madeddu

**Affiliations:** ^1^Division of Experimental Cardiovascular Medicine, School of Clinical Sciences, Bristol Heart Institute, University of BristolBristol, UK; ^2^Congenital Heart Surgery, School of Clinical Sciences, Bristol Heart Institute, University of BristolBristol, UK

**Keywords:** congenital heart disease, scaffold, stem cells, tissue engineering, biomaterial

## Abstract

This review article reports on the new field of stem cell therapy and tissue engineering and its potential on the management of congenital heart disease. To date, stem cell therapy has mainly focused on treatment of ischemic heart disease and heart failure, with initial indication of safety and mild-to-moderate efficacy. Preclinical studies and initial clinical trials suggest that the approach could be uniquely suited for the correction of congenital defects of the heart. The basic concept is to create living material made by cellularized grafts that, once implanted into the heart, grows and remodels in parallel with the recipient organ. This would make a substantial improvement in current clinical management, which often requires repeated surgical corrections for failure of implanted grafts. Different types of stem cells have been considered and the identification of specific cardiac stem cells within the heterogeneous population of mesenchymal and stromal cells offers opportunities for *de novo* cardiomyogenesis. In addition, endothelial cells and vascular progenitors, including cells with pericyte characteristics, may be necessary to generate efficiently perfused grafts. The implementation of current surgical grafts by stem cell engineering could address the unmet clinical needs of patients with congenital heart defects.

## Introduction

Congenital heart disease (CHD) is defined as an abnormality in heart structure that occurs before birth, while the fetus is developing (Sun et al., 2015). It represents the most common congenital anomaly in newborn babies, with a reported prevalence ranging from 6 to 13 per 1000 live births. In the UK alone there are ~4600 babies born with CHD each year (Tennant et al., 2010; Khoshnood et al., 2012). Despite considerable progresses in surgical techniques and medical management of newborns with CHD, there are still considerable mortality and morbidity associated with severe forms of CHD, which comprise the first cause of mortality by congenital abnormalities (Khoshnood et al., 2012).

In the last decade, clinical needs of CHD have extended to the adulthood. Recent estimations indicate that 80% of neonates and infants with CHD can expect to reach adulthood (Woodward, 2011; Khoshnood et al., 2012). According to the Department of Health, in 2006 there were around 135,000 adults living in England with CHD (http://webarchive.nationalarchives.gov.uk/). The 32nd Bethesda Conference report estimated that there were approximately 2800 adults with CHD per 1 million population and that more than half of them have a moderate or high complexity defect (Baumgartner et al., 2010). These patients often develop heart dysfunction and failure as well as neurological, respiratory and coagulation problems (*British Heart Foundation Statistics Database: www.heartstats.org*). The economic and social burden of CHD is high and rapidly increasing. In 2004, the U.S. hospital costs for CHD totaled $1.4 billion (Henaine et al., 2012).

**Figure 1:**
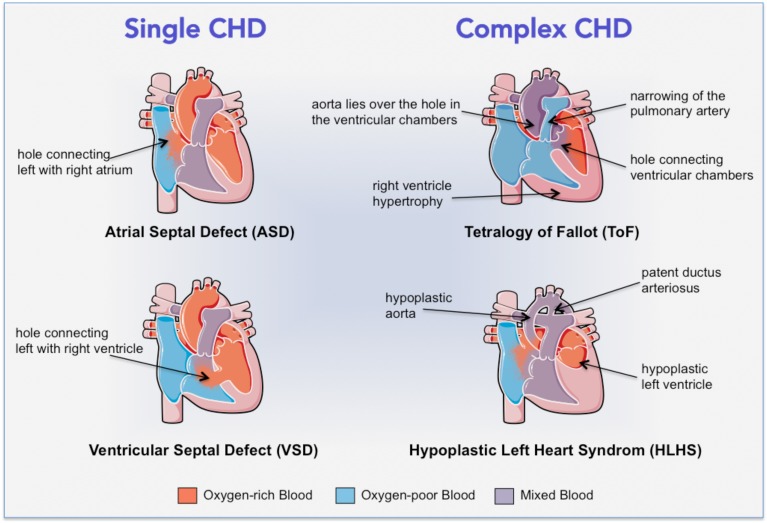
**Cartoon illustrating the cardiac structural alterations in common single and complex CHD**.

Typical congenital abnormalities comprise valves defects, atrial and ventricular septa defects, stenosis and alterations of the aorta and pulmonary veins and arteries and heart muscle abnormalities. The defects can range in severity from relatively simple problems, such as holes between chambers of the heart that can be surgically closed, to very severe malformations, such as the complete absence of one or more chambers or valves, which cause deficits in blood oxygenation and circulation, heart failure (HF), and eventually premature death (Woodward, 2011; Sun et al., 2015). Common *single CHD* are represented by holes (intra-cardiac shunts) inside the internal wall of the heart (Figure 1, left): in *septal defects*, oxygenated blood returning from the pulmonary veins flows from the left to the right chambers of the heart, where it mixes with deoxygenated blood returning from the body, finally causing an overloading of the right ventricle (RV) (Geva et al., 2014; Sun et al., 2015). With time this overload induces remodeling of pulmonary vasculature and hypertension with consequent inversion of the shunt and cyanosis (Woodward, 2011; Sun et al., 2015). When mixed lesions are present at the same time the pathology increases in severity and results in *complex CHD* (Figure 1, right). The most common one is *Tetralogy of Fallot (ToF)*, which features four cardiac abnormalities: narrowing of the pulmonary outflow tract, a hole connecting the ventricular chambers, RV hypertrophy, and the aorta that lies over the hole between ventricular chambers. In ToF patients, an outflow obstruction prevents the blood flowing from the RV into the pulmonary artery, causing deoxygenated blood to pass through the ventricular hole into the left ventricle (LV) and then into the aorta (Woodward, 2011; Wald et al., 2014). Another very serious condition is the *hypoplastic left heart syndrome (HLHS)* (or univentricular heart syndrome), characterized by hypoplasia of the LV, the aorta and related valvular components, with systemic flow becoming dependent on a patent ductus arteriosus. In this condition, blood returning to the heart from both the systemic circulation and the lungs mixes before being pumped by the RV to both the systemic and pulmonary circulation, causing severe cyanosis, increased pressure workload and ultimately failure of the RV (Barron et al., 2009).

This review focuses on stem cell therapy and tissue engineering as a new option to implement current surgical methods for definitive correction of CHD. The approach was initially conceived with the objective to repair and/or replace damaged tissues and organs. However, stem cells from young individuals possess superior naivety and plasticity than adult stem cells and could be better suited for regenerative purposes. The use of scaffolds engineered with stem cells may offer unprecedented therapeutic opportunities for addressing unmet clinical needs of patients with complex cardiac defects.

### Elective surgical correction

The ideal therapeutic option for CHD patients is one-step corrective surgery, during which the heart surgeon closes holes in the heart with stitches or a patch, repairs or replaces valves, widens arteries, and restores the proper location of major blood vessels (Sun et al., 2015). In patients with ToF, the definitive goals are relief of all obstruction to blood flow from the RV to the pulmonary artery and closure of the ventricular septum defect. Reconstruction of RV outflow tract (RVOT) obstruction may involve resection of obstructing muscle bundles, creation of an RVOT patch, pulmonary valvotomy or valvectomy, and pulmonary arterioplasty (Henaine et al., 2012). However, complex CHD usually require more than one open-heart surgery to correct the structural alterations (Woodward, 2011). On the one hand, palliative procedures may be indicated to relieve symptoms of acute HF, allowing definitive correction to be performed when the baby has gained weight and hemodynamics are stabilized (Yuan and Jing, 2009). For instance, babies with HLHS require a surgical palliation within few days from birth as the risk of death is 95% within few weeks from birth without any treatment (Barron et al., 2009; Frescura and Thiene, 2014; Ishigami et al., 2015). On the other hand, multiple re-interventions become often necessary because of deterioration of the implanted grafts (Said and Burkhart, 2014). Patients at the highest risk of death and not suitable for reparative or palliative surgery are candidate to heart transplantation, this extreme option being limited by shortage of donors (Razzouk and Bailey, 2014; Hsu and Lamour, 2015; Ishigami et al., 2015; Sun et al., 2015).

### Limitations of current surgical approach

The use of prosthetic materials in the form of conduits, patches and new valves made by xenografts, homografts, or autografts is routine in congenital cardiac surgery. Even though these grafts may be life-saving, they are characterized by some limitations, represented by a limited durability, and the risks of infection, host immune response, and thrombotic complications. A crucial problem still to be overcome in the pediatric population is the lack of growth and remodeling potential of the grafts currently used for CHD surgery (Mirensky and Breuer, 2008). In the following paragraphs, we illustrate the advantages and disadvantages of clinically available grafts. Additionally, these aspects will be reconsidered in the perspective of creating cellularized scaffolds in a subsequent section of this review.

**Figure 2:**
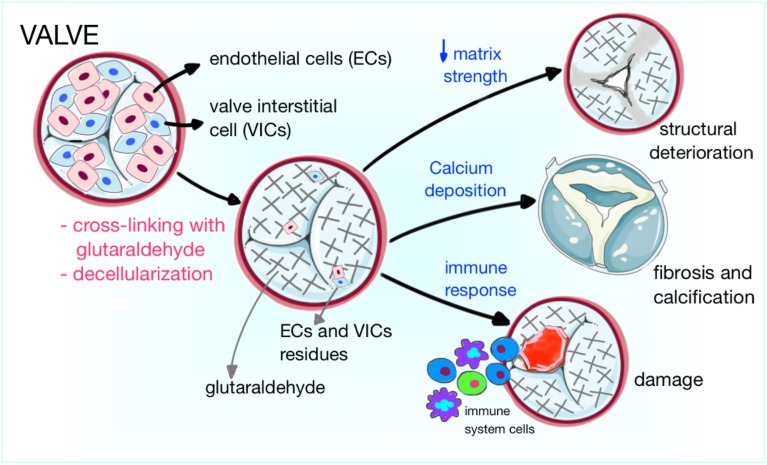
**Cartoon illustrating the mechanisms of prosthetic valve degeneration**. Xenogenic or allogenic valves are decellularized to reduce the risk of immune response and rejection. In addition, animal derived valves can be cross-linked with glutaraldehyde. The three main mechanisms of valve failure are structural deterioration, fibrosis and calcification, and damage by rejection from the host immune system.

*Xenografts* are biological grafts deriving from animals, commonly porcine and bovine, largely used in surgery because of the shortage of human substitutes. Xenogenic bovine pericardium and porcine valves remain the first choice for heart valve substitution (Yap et al., 2013). The porcine valve has the advantage of adequate anatomic structure and unlimited availability. The bovine pericardium contains a higher amount of layered structural proteins than autologous human pericardium, giving it elastic properties that allow conformity to complex anatomical geometries, optimal for RVOT reconstruction (Pok and Jacot, 2011; Strange et al., 2015). In order to avoid the activation of the recipient's immune response, xenografts are decellularized, a process during which animal cells are removed from the graft while the extracellular matrix is preserved, to provide the remaining scaffold with the original anatomical structure (e.g., valve) and the adequate support for the recolonization by the patient's cells after implantation. Different methods have been used for this purpose, among which there are enzymatic cell lysis (Trypsin), detergents and chemicals cell removal (Sodium dodecyl sulfate, Sodium Azide, and Sodium deoxycholate), freeze drying, and a combination of chemical and enzymatic methods (Rieder et al., 2004; Schmidt et al., 2007; Tudorache et al., 2007). An additional type of xenograft manufacture includes cross-linking with chemicals (such as glutaraldehyde), a process by which proteins are cross-linked and collagen fibers stabilized, conferring the graft with tensile strength, elasticity and resistance to degeneration; due to their cytotoxicity, these chemicals eliminate also xenogenic cells (Schmidt et al., 2007; Butcher et al., 2011). However, improvements in pliability and tolerogenicity come at a price (Figure 2). In fact, elimination of valve interstitial cells (VICs), which normally ensure the regular turnover of the valve extracellular matrix (ECM), makes prostheses more susceptible to degeneration, both *in vitro* and after implantation in a mechanical environment. The seeding of VICs onto the decellularized scaffolds has been proposed to overcome this problem; furthermore, the reseeding of the graft using cells able to produce new ECM is crucial to prevent the degeneration of the graft structure (Cushing et al., 2005). Noteworthy, while decellularized grafts are metabolized and remodeled by matrix-metalloproteinases (MMPs) after implantation in the patient, cross-linked grafts do not allow MMPs degradation, thus interfering with the remodeling process (Schmidt et al., 2007). In fact, to preserve the mechanical properties of biological grafts, the remodeling process should be balanced between the matrix formation and degradation. Last, atherosclerotic processes also participate in prosthetic valve remodeling, with initial accumulation of oxidized low-density lipoproteins, followed by monocyte recruitment, generation of a pro-inflammatory milieu, collagen disruption and osteogenic differentiation of resident ECs and precursor cells recruited from the circulation (Shetty et al., 2009; Gossl et al., 2012).

*Cryopreserved homografts*—usually valves or vascular conduits—are derived from humans, commonly cadavers. They are the heart valve replacements closest to the natural valve, being non-thrombogenic and having a low risk of infection. Cryopreserved homografts have been used for many years in CHD surgery, for pulmonary or aortic valve replacement, or for RVOT reconstruction during the Ross operation (Gabbieri et al., 2007; Neumann et al., 2013). Homografts are not chemically cross-linked and exhibit good mechanical properties. Disadvantages are their limited availability, more difficult implantation techniques and failure associated with a specific immune response, especially in young individuals. Although homografts have been associated with a very good hemodynamic performance, the cryopreservation and the thawing process can produce the structural deterioration of the matrices, limiting the durability of the graft (Neumann et al., 2013). In the younger patients, the risk of structural valve deterioration ranges from 71 to 87% at 10 years (Ruel et al., 2005).

*Decellularized homografts* have been proposed as a good alternative, showing lower explantation and degeneration rates than conventional cryopreserved valve grafts (0–10% vs. 30% after 5 years, respectively) (Cebotari et al., 2011; Ruzmetov et al., 2012). Additionally, implantation of decellularized homografts is reportedly associated with spontaneous recellularization (da Costa et al., 2005; Dohmen et al., 2006b; Cebotari et al., 2011).

*Autografts* are created using the patient's own tissues. Pulmonary autografts are commonly used in children and young adults with ToF or valves defects. During the Ross procedure, the surgeon replaces the aortic valve with the autologous pulmonary valve of the patient, and performs the reconstruction of the RVOT using a prosthetic valve (Al-Halees et al., 2002). A study in 30 children demonstrated the growth of the pulmonary autograft in parallel with somatic growth (Simon et al., 2001). However, autografts are prone to dilatation with subsequent aortic valve regurgitation (Dohmen et al., 2002). The freedom from RVOT replacement for children is reported to be about 90% at 12 years (Pasquali et al., 2007). Independently of the material used, *prosthetic reconstruction of RVOT* is often associated with mechanical and electrical abnormalities arising from non-contractile/non-conductive patch material (Tweddell et al., 2000; Perri et al., 2012).

*Autologous pericardium* is easily harvested during cardiac surgery, but is difficult to handle since it has a propensity to roll at the edges rendering the procedure difficult and time-consuming (Pok and Jacot, 2011).

*Synthetic Gore-Tex* and *mechanical valves* are durable but lack of growth potential; moreover, mechanical valves require chronic anticoagulation treatment to reduce the risk of thrombosis. Careful monitoring of coagulation parameters is necessary to avoid bleeding complications (Said and Burkhart, 2014).

Considering limitations and limited durability of available prostheses, there is an urgent need of new therapeutic strategies to optimize long-term outcomes in CHD patients. Recent advances in regenerative medicine allow the manipulation of a spectrum of stem/progenitor cells, including endothelial, mesenchymal, and cardiac stem cells, to support the youngest heart repair. In the following sections, we will report two main approaches of regenerative medicine, namely stem cell therapy (transplantation of dispersed stem cells) and tissue engineering (use of grafts functionalized with single or multiple stem cell types). Stem cell therapy has provided first proof-of-concept, thus paving the way to tissue engineering to fabricate cellularized valves endowed with anti-coagulative properties and living cardiac tissue that grows physiologically in parallel with the heart's growth.

## Stem cell therapy for CHD

Cell therapy holds promises for treatment of a range of disabling diseases. This therapeutic strategy is based on the injection of dispersed cells in the site of damage, aimed at stimulating the regeneration of the damaged tissue and possibly the recovery of its function. In the heart, different ways of delivery can be used, that are intracoronary, intramyocardial, intravenous, and epicardial. The therapeutic effects can be determined by a direct cellular effect, given by transdifferentiation of injected cells in cardiovascular cells, or more likely by a cytokine-paracrine effect, that means cells release soluble factors that exert protective effects on neighboring cells, although not participating in the formation of new functional tissue by trans-differentiation (Krause et al., 2010). From recent studies a new paracrine mechanism has emerged, based on the release of intracellular material—mainly proteins and micro-RNA—by particular vescicles called exosomes (Ibrahim et al., 2014).

In the cardiovascular medicine field, stem cell-based therapies have been principally applied to the treatment of adult patients with myocardial ischemia or HF (reviewed in Sanganalmath and Bolli, 2013). The first two randomized phase I clinical trials, SCIPIO and CADUCEUS, in which respectively c-Kit-positive Cardiac Stem Cells and Cardiosphere-derived Cells were administered to end-stage HF adult patients, showed positive outcomes regarding safety and efficacy of cell therapy (Bolli et al., 2011; Makkar et al., 2012). Only recently, the approach has been proposed for correction of congenital cardiac defects (Bernstein and Srivastava, 2012; Pincott and Burch, 2012).

### Which cell type should be used?

A comprehensive solution for complex cardiac defects like ToF requires the generation of valves and grafts containing a spectrum of regenerative cells. On the one hand, the use of multipotent stem cells able to generate all the components of the cardiac tissue, i.e., vascular cells, cardiomyocytes, and stromal cells, represents an option. On the other hand, the combination of different progenitor cells with restricted and predictable lineage commitment might be preferred by regulatory agencies for safety reasons. Hence, at this stage, research on different cell types should be pursued.

#### Induced pluripotent stem cells

Induced pluripotent stem cells (iPS) are pluripotent cell lines obtained from the *ex vivo* reprogramming of fetal or adult somatic cells, like fibroblasts. During the reprogramming process, commonly four classical recognized pluripotency factors (NANOG, OCT-4, c-MYC, and KFL-4) are introduced within the cells from an ectopic source, usually carried by retroviral vectors. Reprogrammed cells are characterized by a high plasticity, being able to differentiate in cells of the three embryonal germ layers and thus potentially having the capacity to differentiate in most of the human body's cells (Takahashi et al., 2007; Yu et al., 2007). These last properties make iPS a good choice for application in regenerative medicine. For employment in CHD surgery, iPS derived from the patient's cells could be ideally differentiated *in vitro* in vascular and myocardial cells before autologous transplantation (Lundy et al., 2013; Yoder, 2015). To date, the limitation to the clinical use of these cells is given by the risk of tumorigenesis deriving from the genomic integration of the viral vectors (Mayshar et al., 2010).

#### Foetal and umbilical cord cells

The time of diagnosis is key for the decision. Owing to advances and diffusion of pre-natal cardiac imaging, it is now possible to recognize cardiac defects in a large proportion of subjects. For these cases, foetal cells and umbilical cord cells represent valid therapeutic candidates and, in the future, *in utero* repair of cardiac defects using these cells will become a routine practice. Umbilical cord is collected at the time of birth; umbilical cord blood mononuclear cells (UCBMNCs) can be isolated from the blood, while mesenchymal stem cells are extracted from the Wharton's jelly. Cord stem cells are able to differentiate in cardiomyocyte-like cells and endothelial cells (ECs) (Chen et al., 2009; Wu et al., 2009). Last year, the Mayo Clinic announced the first U.S. stem cell trial with autologous umbilical cord blood cells to treat children with HLHS (http://www.mayo.edu). Foetal-derived stem cells can be isolated from the amniotic fluid and include both pluripotent stem cells and more committed cells (Klemmt et al., 2011). Foetal cells could be stored, according to the large experience accumulated with umbilical cord blood cells, and used for multi-stage corrections.

#### Adult cells

Despite the promising potential of iPS and foetal cells, so far most preclinical and clinical studies have employed post-natal cells for both safety reasons and easy accessibility to adult tissues. Different cell types derived from post-natal tissues can be used.

##### Endothelial cells and progenitor cells

Initial focus of research has been the re-endothelialization of cardiac valves to reduce thrombotic complications. Optimal candidates for this purpose are ECs and endothelial progenitor cells (EPCs). The first can be isolated from peripheral, easy accessible veins, such as the saphenous or forearm veins, while the second ones can be purified from the peripheral blood or bone marrow. EPCs can circulate in peripheral blood and can be incorporated in regions of active neovascularization, such as the ischemic myocardium (Xin et al., 2008). Experimental evidence suggests that EPCs participate not only in the process of vasculogenesis substituting the lost ECs but also in the endothelialization of grafts (Young et al., 2007). The significance of EPCs in cardiovascular disease has been reviewed in Madonna and De Caterina (2015).

##### Mesenchymal stem cells

Mesenchymal stem cells (MSCs) are a heterogeneous subset of stromal stem cells that can be isolated from many adult tissues, including the heart, skeletal muscle, bone marrow and adipose tissue (Uccelli et al., 2008). MSCs stand out as an encouraging option for cell therapy due to their accessible isolation, great expansion potential, immunoregulatory activity and angiogenic properties (Dimarino et al., 2013). Not less relevant, MSCs possess a multipotential differentiation capacity, being able to differentiate, *in vitro*, into cells of the mesodermal lineage (Dominici et al., 2006), and possibly toward cells of endoderm and ectoderm derivation (Uccelli et al., 2008). *In vivo*, although being able to generate new cardiomyocytes and vascular cells, MSCs stimulate vascular and cardiomyocyte regeneration acting prevalently by paracrine mechanisms (Gnecchi et al., 2006, 2012). Also, they may positively influence cardiac metabolism and contractility (Gnecchi et al., 2012). The immune compatibility of the MSCs is a remarkable advantage for the translation of their use in clinics (Castro-Manrreza and Montesinos, 2015).

##### Bone marrow-derived progenitor cells

Total bone marrow-derived cells (BMCs) and especially subpopulations of bone marrow mononuclear cells (BMMNCs, that means either MSCs, or hematopoietic stem cells (HSCs), or monocytes) have been used in transplantation trials in acute myocardial infarction (MI) patients (reviewed in Simari et al., 2014). The advantage of using these cells is the easy accessibility with low risks for the patients, and the possibility to harvest high numbers of cells without requirement of long time *in vitro* expansion. The mechanisms by which BMCs can contribute to an improvement of cardiac function after transplantation in the patient heart are still debated, but two main mechanisms have been proposed: (1) paracrine influence on surrounding cardiac cells (Uemura et al., 2006), and (2) variable levels of transdifferentiation in ECs, pericytes, VSMCs and cardiomyocytes (Hosoda et al., 2010; Yoon et al., 2010). Noteworthy, the potential of transdifferentiation in cardiomyocytes still represents an open challenge for stem cell scientists; regarding the BM-MSCs subpopulation, there are discrepancies between scientists who reported the cells ability to generate new cardiomyocytes *in vivo* post-MI (Kawada et al., 2004; Yoon et al., 2005) and those who showed, instead, that BM-MSCs have very limited ability to generate functional contractile cardiac cells (Rose et al., 2008; Beitnes et al., 2009; Dixon et al., 2009; Tendera et al., 2009; Wohrle et al., 2010). Conversely, this cardiogenic ability has been negated to HSCs (Balsam et al., 2004; Murry et al., 2004).

##### Cardiac stem cells

Harvesting MSCs from accessible sources, like the subcutaneous adipose tissue, represents an attractive option. However, MSCs present in the stroma of different tissues are heterogeneous and may be influenced by the specific environment where they reside. The idea of using more specialized cell populations was pursued with the use of MSCs obtained from the target organ: the heart. Several investigations over the last 15 years have demonstrated that new cardiovascular cells are generated starting from primitive undifferentiated cells (*cardiac stem cells—CSCs*) resident in the heart since from birth, with this phenomenon playing a primary role after an acute injury as a myocardial infarction (Torella et al., 2006; Bearzi et al., 2007; Hsieh et al., 2007; Bergmann et al., 2009). Several CSC classes have been identified in the adult human heart, based on the expression of different markers (Torella et al., 2007; Beltrami et al., 2011; Bernstein and Srivastava, 2012). The most extensively characterized CSCs express the Stem Cell Factor-receptor (SCF-R, also named c-Kit), the Stem Cell Antigen-1-like (Sca-1) and the Multidrug Resistance-1 (MDR-1, receptor which belongs to the class of ABC transporters that mediate the Hoechst dye efflux from the cell) (Muller et al., 2002; Quaini et al., 2002; Messina et al., 2004; Bearzi et al., 2007). All the above cells do not express transcription factors or membrane and cytoplasmic proteins shared by mature cell types, and thus they are defined as Lineage-negative (Lin-); moreover CSCs are characterized by the capacity of self-renewal, clonogenicity, and multipotency, being able of differentiation in all the mature cardiovascular cell types. All the mentioned properties are essential for the formation of new cardiac tissue, although this latter phenomenon is not sufficient to restore a massive loss of tissue (Muller et al., 2002; Quaini et al., 2002; Messina et al., 2004; Bearzi et al., 2007).

##### Combinatory cell therapy

The heart is made by different types of cells. Therefore, optimum cell therapy for cardiac repair may require the association of different cell populations with complementary activities leading to balanced cardiomyogenesis/vasculogenesis. Apart from a recent study reporting the benefit of associative treatment with human MSCs and human c-Kit+ CSCs in an immunosuppressed swine model of myocardial infarction (Williams et al., 2013), combinatory cell therapy has received very little attention. We recently reported the advantage of dual therapy with CSCs and vascular pericytes for harmonized repair of the infarcted heart in mice (Avolio et al., 2015a). Interestingly, the two cell populations exert reciprocally enhancing paracrine activities, which lead to increased proliferation of vascular cells and cardiomyocytes and attraction of endogenous CSCs. These seminal examples of combinatory cell therapy set the basis for fabrication of scaffolds functionalized with multiple cell types.

##### Stem cells in the youngest heart

Despite minor attention has been focused on the pediatric heart, a few studies displayed that CSCs are more abundant in the neonatal period and rapidly decrease over time, making myocardial samples from the youngest hearts an optimal source of stem cells for use in reconstructive surgery (Peral et al., 2014). In particular, the histological examination of RV biopsies from CHD patients aged 2–93 days demonstrated that c-Kit-positive CSCs are four-fold more abundant during the first post-natal month (0.4 vs. 0.1% of total cardiac cells at birth and after 1 month, respectively), and a similar reduction was observed also for NKX2.5-positive cells (Amir et al., 2008). Moreover, Mishra et al., compared c-Kit-positive CSCs isolated from right atrial (RA) specimens of CHD patients selected on the basis of 3 different groups of age: neonates (<30 days), infants (1 month to 2 years) and children (2–13 years), and showed that c-Kit-positive CSCs are two- and three-fold higher in neonates than in infants and children respectively, along with a superior differentiation potential (Mishra et al., 2011). Similarly, a comparison of human hearts aged between 9 days to 77 years showed that the number of CSCs isolated per mg of cardiac tissue was more than three-fold higher in the youngest hearts (Tateishi et al., 2007). In addition to these considerations, a study performed in children aged 19 days to 16 years affected by univentricular heart and chronic pressure overload, showed that the number of c-Kit-positive cells in RV of patients is three-fold higher than in cardiac biopsies post-transplantation, suggesting that the pressure overload may lead to an increase in resident CSCs (Rupp et al., 2012a). This latter may represent an adaptive response in CHD children in the attempt to counteract the alterations in cardiac function, although not sufficient to overcome the challenges of severe CHD.

But stem cells resident in neonatal hearts are not only more abundant than the adult counterparts. A study performed by Simpson et al., showed that c-Kit/CD90-positive CSCs generated from neonatal RA samples are also endowed with a superior regenerative ability than CSCs derived from adult samples, when transplanted in a rat model of acute myocardial infarction (Simpson et al., 2012). Authors showed that neonatal CSCs are characterized by higher levels of c-Kit, flk-1 and Islet-1-positive cells in comparison with the adult ones; the younger cells possessed also an increased cardiomyogenic potential both *in vitro* and *in vivo*, preventing the formation of the fibrotic scar and improving cardiac functional recovery when transplanted in the animal hearts. Authors supposed that a possible explanation for this augmented regenerative ability of neonatal CSCs might derive from the higher secretion of pro-angiogenic factors, like Vascular Endothelial Growth Factor (VEGF) and Angiogenin, which play a crucial role during the formation of new blood vessels (Simpson et al., 2012).

Besides the above mentioned CSC classes, other cell types have been isolated from the youngest human heart, and may represent a valid option in cell therapy for correction of CHD. Very recently, a population of heart pericytes (HPs) has been identified by the group of B. Péault in myocardial samples from both fetuses at 17–23 weeks of development and post-mortem adults. HPs, recognized as CD34/45-negative and NG2/PDGFR-β/CD146-positive cells, possess a multilineage mesodermal differentiation potential, including limited cardiomyogenic properties both *in vitro* and *in vivo* in a mouse model of MI, and marked pro-angiogenic ability *in vitro* (Chen et al., 2015). Despite this, the potential of these cells has not been evaluated immediately after birth yet. Instead, our group has recently been involved in the isolation and characterization of a population of cardiac pericytes (CPs) from myocardial leftovers of neonates and children operated for correction of CHD (Avolio et al., 2015b). We originally select CPs as CD34-positive CD31-negative cells, and after expansion CPs express pericyte (NG2/PDGFR-β) along with stemness markers (NANOG, OCT-4, SOX-2), are clonogenic and endowed with ability to differentiate into VSMCs, but not into ECs or cardiomyocytes. We demonstrated that CPs are able to attract endothelial and cardiac stem cells in an *in vitro* migration assay, and support the network forming ability of ECs in an *in vitro* angiogenic assay (Avolio et al., 2015b). Last, in the human fetal aorta a population of CD133/CD34/VEGFR-positive vascular progenitor cells has been identified and characterized for its unique vasculogenic and myogenic potential in a mouse model of limb muscle ischemia (Invernici et al., 2007).

### Preclinical trials

Since the complex nature of congenital heart defects makes these latter difficult to reproduce in animals, there are no many animal models of CHD, and this represents a significant limit in the translation of new therapies to clinic (Peral et al., 2014; Tarui et al., 2014). Nevertheless, in the last years some efforts and progresses have been done, in particular to reproduce an increased pressure or volume overload of the RV that is a common feature in patients with a HLHS or ToF. Although it is difficult to make an animal model that mimics univentricular hearts, a pressure-overload right-heart model using pulmonary artery banding has been developed in rats (Hoashi et al., 2009) and sheep (Davies et al., 2010), which is useful to evaluate the safety and efficacy of cell transplantation in presence of single ventricular lesions. In these studies, skeletal myoblasts (Hoashi et al., 2009) and cord blood stem cells (Davies et al., 2010) were injected in the animal hearts, determining an improvement of RV function. Also, a first murine model of RV volume overload has been obtained by Reddy et al., by entrapping the pulmonary valve leaflets with sutures (Reddy et al., 2013). In addition, a recent study performed in a piglet ToF model showed the safety and feasibility of intramyocardial administration of human MesP1 (mesodermal posterior 1)-positive /SSEA-1(stage specific embryonic antigen-1)-positive embryonic stem cell-derived cardiac progenitors 4 months after a surgical procedure mimicking the repair of ToF. This latter consisted of an enlargement of the RVOT by a polytetrafluoroethylene patch, the excision of one pulmonary valve leaflet and a pulmonary artery banding. At 3 months follow-up, animals receiving cell therapy showed some improvements in RV remodeling (reduction of peri-myocyte fibrosis) but unfortunately no significant improvement in RV function compared to control animals (Lambert et al., 2015). Finally, the feasibility and long term safety of autologous UCBMNCs transplanted intramyocardially into the RV of piglet hearts was recently demonstrated (Cantero Peral et al., 2015).

### Clinical trials in children with CHD

So far, major attention has been dedicated to the therapy of pediatric dilated cardiomyopathy (Rupp et al., 2009; Bergmane et al., 2013; Selem et al., 2013), while experience with stem cell therapy in children with severe CHD or acquired heart failure has been limited to single cases and small-size cohorts.

In 2010, Rupp et al., reported a case of cell therapy with intracoronary injection of autologous BMCs in an 11-months-old boy with HLHS; although this was only an isolated case, the patient outcome was dramatically improved 3 months after cell therapy, with RV ejection fraction (RVEF) increasing from 22 to 44% (Rupp et al., 2010). More recently, intraoperative administration of autologous UCB-stem cells in a 4-months-old baby undergoing a second palliation surgery gave positive results, improving the RVEF from 30 to 50% at 3 months of follow-up (Burkhart et al., 2015). Again, another study performed in 9 children with severe terminal HF (6 with HF secondary to dilated cardiomyopathy and 3 with CHD) showed the feasibility and safety of intracoronary infusion of autologous BMMNCs, stabilizing patients conditions during the short (3 months) and long term (up to 52 months) follow-up (Rupp et al., 2012b).

The first long term follow-up phase I controlled clinical trial in pediatric patients with CHD, using autologous CSCs, has been concluded only recently (Ishigami et al., 2015). In the TICAP trial (Transcoronary infusion of Cardiac progenitor Cells in patients with single ventricle physiology), autologous CSCs were isolated from 7 children affected by HLHS, aged 5 months to 3 years, and administered via intracoronary delivery 4–5 weeks after surgical palliation. Seven children were selected as control (received palliation surgery without cell therapy). The administration procedure was safe, without any serious adverse event. At 18 months of follow-up, the CSC-treated patients demonstrated an improvement in RVEF from an average baseline value of 47 to 54%, whereas control patients showed little improvement in RVEF, from 47 to 49% (Ishigami et al., 2015). This study gave the first important demonstration of safety and feasibility of autologous CSCs application in young CHD patients. The same group is now carrying out a larger phase II study (Cardiac progenitor Cell infusion to treat univentricular heart disease: PERSEUS), which involves 34 patients randomly assigned to the treatment or control group, to strengthen the positive results collected during the first phase (Tarui et al., 2014).

## Tissue engineering for CHD correction

Tissue engineering is a promising bioengineering technology that aims at the creation of unique substitutes using three-dimensional synthetic or biologic scaffolds seeded with autologous stem cells and differentiated cells, to allow individual patients therapy with regeneration, remodeling and growth potential (Cheema et al., 2012; Smit and Dohmen, 2015). On the one hand, scaffolds serve as a site of cell attachment and new tissue formation. On the other hand, their recolonization by cells after implantation seemingly contributes to the creation of a living tissue perfectly integrated in the recipient's organ. Seeded cells may contribute to the homing of resident cells by secretion of chemoattractant factors.

**Figure 3:**
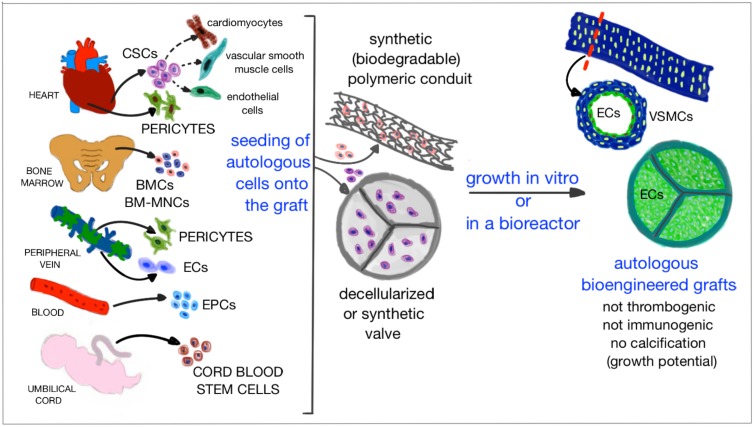
**Cartoon illustrating the promising strategy of tissue engineering based on which grafts and materials are combined with patient autologous cells and grown** ***in vitro*** **or in a bioreactor, in order to obtain an optimized cellularized graft that lacks of immunogenicity, thrombogenicity, and risk of calcification, while having the potential to grow in parallel with the child growth**.

Tissue engineering has been one of the most promising strategies for the regeneration of impaired tissues. For instance, a tissue engineering approach has been used successfully for the repair of injuries to, or congenital absence of complex organs such as the trachea, esophagus, or skeletal muscle (Macchiarini et al., 2008; Badylak et al., 2012). The first success, in 1994, was the reproduction of tracheal cartilage using tissue engineering techniques (Vacanti et al., 1994). Later, bioengineering techniques have been applied also to the heart. Experience accumulated in adult patients suggests that dispersed stem cells may die soon after implantation in the heart, their therapeutic action being attributable to paracrine factors released during the initial post-transplantation phase. Therefore, this approach does not seem to be advantageous for the repair of complex congenital defects, that frequently requires additional tissue in various forms, such as patches, valves and conduits (Dean et al., 2012; Kalfa and Bacha, 2013; Smit and Dohmen, 2015). Stem cells may work more efficiently if embedded in extracellular matrices, prosthetic grafts and patches to create biological structures that, once implanted in the defective heart, can grow and remodel in a physiologic manner in parallel with cardiac and whole body growth (Figure 3) (Bertipaglia et al., 2003; Scholl et al., 2010).

### Which characteristics should an optimal bioengineered graft have?

The creation of an optimal bioengineered graft relies upon the careful choice of two main protagonists: (1) the biomaterial and (2) the cells that will repopulate the biomaterial graft. Scaffolds used in cardiac surgery should provide structural integrity at the time of repair, while permitting infiltration of cells from native tissue and enabling the development of new vessels and blood supply, which contributes to tissue remodeling. The cell-scaffold constructs are configured to serve as a template to promote development of structures that closely mimic the original tissues (Cheema et al., 2012; Fallahiarezoudar et al., 2015). Interestingly, physiological cyclic strain and shear stress may even accelerate the formation of tissue starting from the cellularized graft (Engelmayr et al., 2006). The final result should be a new tissue composed entirely of the patient's autologous cells, able to remodel in response to physiological changes (Butcher et al., 2011; Dean et al., 2012). Not less important, an advantage of coating the prosthetic materials with stem cells or ECs before transplantation is the prevention of thrombotic complications.

The optimal reparative patch for closure of atrial and ventricular septal defects and RVOT reconstruction, as well as the optimal substitutive valves used in pediatric CHD, ideally should possess the following characteristics (Butcher et al., 2011; Dean et al., 2012; Kalfa and Bacha, 2013; Alsoufi, 2014):

- absence of immunogenicity,
- growth potential proportional to somatic enlargement,
- excellent durable hemodynamic profile,
- availability in different sizes,
- pliability,
- association with minimal thromboembolism risk thus not requiring anticoagulation,
- low incidence of structural degeneration,
- resistance to calcification.

These characteristics are critical especially in newborns, in which corrections should be durable for all the course of a normal lifespan, to avoid repeated risky operations. But to date, the optimal scaffold still does not exist, although many progresses have been done in this direction, and a wide series of biological and synthetic materials are currently under evaluation in the attempt to find the optimized one.

An important factor to consider during the creation of a bioengineered graft is its mechanical behavior. Before proceeding with any preclinical validation, the performance of bioengineered grafts is first evaluated *ex vivo*. This is done both before and after the seeding of cells, to understand how the cells affect the matrix remodeling in biological grafts. During these tests, the hydrodynamics functions and the durability of the graft are evaluated, both under static conditions and in presence of pulsatile flow. The elasticity and stiffness of the graft are evaluated with both uniaxial and biaxial mechanical tests (Ghanbari et al., 2009).

### Adaptability of currently available grafts to tissue engineering

**Figure 4:**
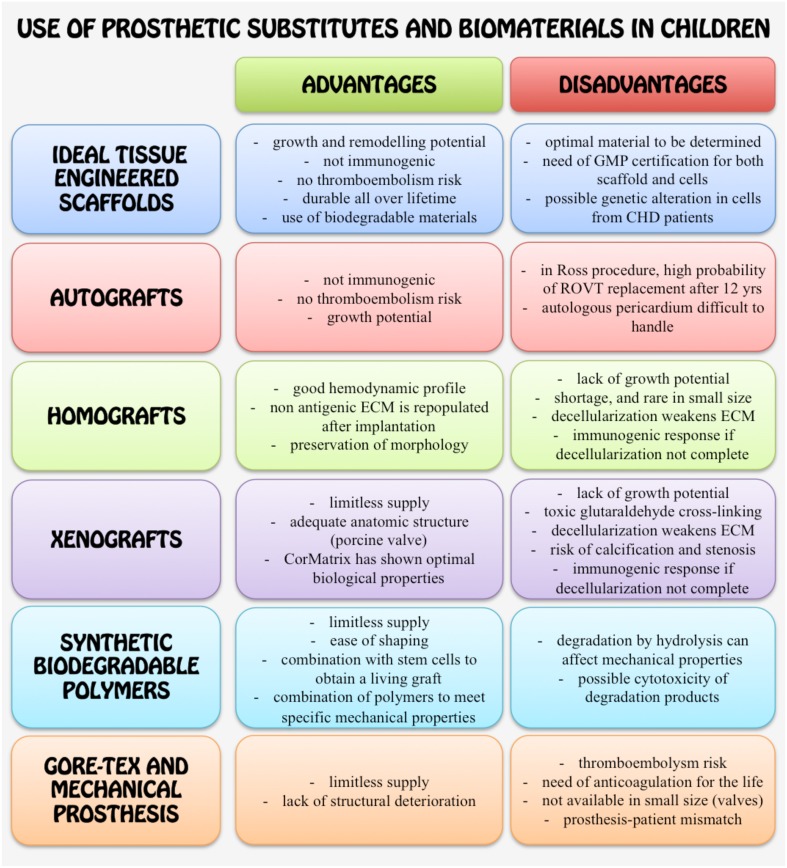
**Schematic cartoon summarizing the main advantages and disadvantages of using the different synthetic or biological materials and grafts for surgical correction of CHD in children**.

Some undesirable characteristics of currently available grafts discourage the application in CHD patients, but fortunately progresses have been done and promising solutions are underway. The main advantages and disadvantages of materials used for correction of CHD are recapitulated in Figure 4.

One matter of concern is that the toxicity of chemical agents used during manufacture of prostheses might inhibit the functionalization by autologous cells. A coating with bioactive substances has been proposed to overcome the last problem and promote the process of recellularization *in vivo* Dohmen et al., 2006a; Butcher et al., 2011; Strange et al., 2015. Moreover, the use of glutaraldehyde has been challenged because of concerns about the promotion of calcification. Calcification of grafts, in particular valves, is a major problem since it can cause the failure of the graft and the occurrence of further surgery. The risk of calcification in children is even higher than in adults, as in young patients the mobilization of calcium, due to bone remodeling, accelerates the calcification process. A possible solution is represented by anti-calcification treatments (Butcher et al., 2011; Pok and Jacot, 2011). It remains unknown if calcification may affect cells used for tissue engineering. This risk may be higher for cells, including MSCs, which have the propensity to differentiate into osteocytes under inductive conditions (Ohata and Ozono, 2014). However, it cannot be excluded that other cell types may not only resist to xenograft manufacture agents but also contrast their pro-calcifying action.

The *CorMatrix*® ECM (CorMatrix Alpharetta, GA), a patch made of decellularized porcine small intestinal submucosa extracellular matrix (SIS-ECM), displays a lot of potential advantages over other materials currently used in pediatric cardiac surgery, such as ease handling and implantability, abundance, low immunogenicity, high mechanical strength, minimal scar formation, recruitment of host cells, remodeling without calcification and possible growth potential (Pok and Jacot, 2011; Kalfa and Bacha, 2013). CorMatrix patches have been successfully used in congenital heart surgery for pediatric cardiac and vascular reconstructions (Scholl et al., 2010; Quarti et al., 2011; Witt et al., 2013). Recently, our group confirmed the great recellularization properties of the CorMatrix, successfully growing cardiac pericytes—isolated from children affected by CHD—onto CorMatrix patches up to 3 weeks in a bioreactor, finally showing the engraftment and viability of cells (Avolio et al., 2015b).

Recently, scaffolds have been fabricated using synthetic *biodegradable polymers*, such as polyglicolyc acid (PGA), poly-L-lactic acid (PLLA) and polycaprolactone (PCL). On one hand, the advantages of using these polymers are a limitless supply, ease of shaping, and the possibility to combine the concentrations of the polymers to generate scaffolds that meet the compliance specifications of the environment in which they have to be introduced. But on the other hand, the degradation process by hydrolysis will affect the mechanical properties over time. The degradation rates have been estimated in 2–3 months for PGA and 2 and 3 years for PLLA and PCL, respectively. Although some concerns have been reported about the cytotoxicity of the products of the degradation process (Butcher et al., 2011; Dean et al., 2012; Fallahiarezoudar et al., 2015), these materials are currently the most used in tissue-engineering procedures for the construction of autologous grafts made by the patient's own tissue.

### Methods of cell seeding and growth of tissues *in vitro*

During the generation of a bioengineered graft, not only the selection of the proper cell type and the choice of a physiological culture method are crucial in order to achieve good results, but also cell seeding is one of the key issues. How to seed cells efficiently and uniformly, especially in the inner parts of scaffolds with pores, and with no impairment to the cells, has been one of the major challenges for tissue engineering.

Since from the beginning, *static seeding* has been the most frequently used because of its simplicity and the ease to be performed within a common cell culture laboratory, since it does not require sophisticated equipment. However, the efficiency of static seeding is always low, and the distribution of the cells throughout the scaffold is not uniform, with the inner part of the scaffold holding few cells, even if excellent biocompatible scaffolds with big pores are used. As a consequence, only the superficial layer of the scaffold will be formed by the regenerated tissue (Dai et al., 2009).

One important factor that limits the penetration of cells inside the scaffold is represented by air in the pores, which explains why static seeding cannot reach satisfactory results in term of scaffold repopulation. Different solutions have been considered to overcome this bottleneck.

In *centrifugation seeding*, a moderate centrifugal force is applied during the seeding process, to facilitate the penetration of cells in the central part of the scaffold (Godbey et al., 2004; Roh et al., 2007). However, whether survived cells remain functional after centrifugation has still to be determined. Moreover, during centrifugation the orientation of the scaffold cannot be controlled, thus that the overlapping of material pieces does not allow a homogeneous distribution of cells. Instead, the *low-pressure seeding* uses a vacuum desiccator to create a reduced pressure that draws the air in the materials out by pressure difference, facilitating the penetration of the culture media and cells inside the pores (Torigoe et al., 2007). However, despite the seeding efficiency is increased, the low pressure may affect cell viability and function after seeding, thus additional investigations are required (Dai et al., 2009). In *perfusion seeding*, a continuous cell suspension perfusion is applied through 3D-scaffold pores using bioreactors to assist in cell infiltration and to aid in nutrition (Dai et al., 2009).

A *bioreactor* is a device able to ensure the biological and physiological environment of the heart and the circulatory system. Mimicking the physiological mechanical forces, shear stress and blood flow, the bioreactor can allow the construction of engineered scaffolds that will be functional after transplantation into the patients (Carrier et al., 1999; Hecker and Birla, 2007). When cellularized scaffolds are kept in culture for long time, the tissue grows and reaches a 3D-structure, so that the static culture system is not adequate to guarantee the perfusion of culture media uniformly. A solution to this problem comes from the dynamic bioreactors, which can maintain viability of tissue-engineered organs by using both mechanical and biochemical conditioning. Apart the basic requirements of cell cultures—which include the control over the time of dissolved O_2_ and CO_2_, pH, temperature and nutrients concentration—a dynamic bioreactor can provide the control of flow waveform and physiological pressure, keeping culture medium under the pulsatile flow that enables the perfusion of the media inside the entire graft and simulates at the same time the *in vivo* pressure and heart rate (Carrier et al., 1999; Hecker and Birla, 2007).

### Preclinical studies and clinical application of tissue engineered-grafts

Tissue engineering-based grafts have been tested in small and large animal models to assess their safety and feasibility before translation to clinics. So far, BMMNCs and ECs have been frequently selected for preclinical studies of tissue engineering applications, probably due to the relative ease of harvesting BM aspirates and peripheral veins compared, for example, to samples of cardiac source. For the sake of brevity and clarity, main animal studies are summarized in Table 1. During these procedures, scaffolds were firstly seeded with cells *in vitro*, and secondly implanted into the animals. The results obtained with animals encouraged the test in humans.

**Table 1:** **Preclinical studies with tissue-engineered grafts**.

**Study**	**Animal model**	**Cells seeded**	**Scaffold used**	**Outcome**
Matsumura et al., 2003b	Dog	Allogenic BMMNCs	Copolymer of LA/CL covered by PLLA	TE-grafts were implanted into the vena cava. After up to 8 weeks, no stenosis was observed and cells on the grafts expressed endothelial and VSMc markers
Vincentelli et al., 2007	Lamb	Allogenic BMMNCs or MSCs	Decellularized porcine pulmonary conduits	TE-grafts were implanted into the pulmonary artery. After 4 months, both the valves were recolonized and re-endothe-lialized. BMMNC-valves were thicker and showed inflammatory cell infiltration, while MSC-valves displayed extracellular matrix and cell disposition close to those of native pulmonary valves
Brennan et al., 2008	Lamb	Autologous BMMNCs	PGA scaffolds covered by LA/CL	TE-grafts implanted as inferior vena cava (IVC) interposition grafts. After 6 months, grafts were patent and increased in volume, with no evidence of aneurysmal dilatation. They were histologically comparable to the native IVC
Roh et al., 2010	SCID/beige mice	Xenogenic human BMMNCs	PGA scaffolds covered by LA/CL	TE-grafts were implanted as inferior vena cava interposition grafts. After 24 weeks the original scaffold was degraded and substituted by organized layers of ECM, endothelial and smooth muscle cells, resembling the native IVC
Sutherland et al., 2005	Sheep	Autologous BM-MSCs	PGA/PLLA	The pulmonary valve was resected and TE-valve was implanted into the pulmonary artery. After 4 and 8 months grafts were histologically comparable to the native valve
Shinoka et al., 1995	Lamb	Allogenic ovine artery fibroblasts and ECs	PGA leaflets	The right posterior leaflet of the pulmonary valve was resected and replaced with a TE-valve leaflet. Absence of stenosis. Development of ECM with appropriate cellular architecture
Dohmen et al., 2006b	Sheep	Autologous ECs from jugular vein	Decellularized valve	Scaffold was implanted into the RVOT. After 6 months, there was no calcification, and histologically ECs and fibroblasts were observed
He et al., 2010	Rat	Xenogenic human skeletal muscle pericytes	Poly(ester-urethane) urea scaffolds	TE-grafts were implanted end-to-end into the abdominal aorta. After 8 weeks, pericytes evenly populated the graft. TE-grafts presented extensive tissue remodeling with organized layers of endothelial and smooth muscle cells, and collagen and elastin, resembling the native arterial conduit

Positive results have been obtained also from clinical studies involving the application of tissue engineering-based grafts in children affected by CHD, although they are still limited to few reports. In 2001, the transplantation of a tissue engineering-based graft on a 4-years-old girl with univentricular heart and pulmonary atresia was recorded. Autologous cells were isolated from a peripheral vein and seeded onto a biodegradable tubular scaffold composed by PLLA/PCL reinforced with PGA, designed in order to degrade within 8 weeks. Seven months after implantation, the conduit was not occluded and there was no evidence of aneurysm (Shin'oka et al., 2001). Two years later the same group reported other clinical cases of children with CHD who received a biodegradable tissue engineering-based graft (PLLA/PCL, designed to be degraded in 3–5 years) seeded with total BMCs or selected BMMNCs. Again, the results were positive, with no stenosis documented in the grafts (Matsumura et al., 2003a). The successful transplantation of a vascular biodegradable conduit (PLLA/PCL reinforced with PGA) seeded with saphenous vein derived cells was recorded in a 12-years-old during the Fontan operation (Naito et al., 2003). Later, the clinical results of the application of tissue engineering-based grafts seeded with autologous BMCs in CHD patients (25 out of 42 patients recruited were <7 years) were published (Shin'oka et al., 2005). The scaffolds were made by PLLA/PCL and PGA, degradable in 2 years. The results were positive, without thrombosis, stenosis or obstruction of the grafts after a median of 16 months; grafts remained patent and their diameter increased in size (Shin'oka et al., 2005). More recently, a similar study was conducted in patients with single ventricle physiology (17 out of 25 patients recruited were <7 years), in which PLLA/PCL/PGA grafts seeded with autologous BMMNCs were implanted as extracardiac cavopulmonary conduits. Four out of 25 patients presented graft obstruction that required angioplasty, but all the other patients experimented a good recovery without failure of the graft (Hibino et al., 2010).

Despite these first encouraging results, the performance of TE-grafts in CHD children still needs to be investigated, since a main limitation of the studies performed so far is the lack of a long term follow-up. Nevertheless, application of tissue engineering-based grafts to the correction of CHD is still a new field of study, which will be extensively expanded in the coming future.

## Future perspectives

The choice of the appropriate cell type to use for cell therapy is of crucial importance to reach the desired therapeutic goal. In the future, cell types able to guarantee on the one hand the regeneration of the missing or lost tissue, and on the other hand the parallel remodeling of the extracellular matrix in order to provide the new generated tissue with the ability to adapt with the anatomy of the growing cardiovascular system, might be privileged; also, combinatory cell products may represent an interesting and safe option. But, while there is space for improvement in the characterization and quality of the cell product, the field of regenerative medicine for treatment of CHD urgently requires tissue engineering solutions, as dispersed cells are not ideal to reconstruct valves and conduits. To date, one major limitation in the use of prosthetic devices is the need of replacement. Research is currently underway to provide biologically compatible solutions. While there is a remarkably growing interest in possibilities offered by cell therapy and tissue engineering, the clinical application in newborns and children is still in its pioneering phase.

Cell therapy products or tissue-engineered products are classified as advanced therapy medicinal products (ATMP) that need to be authorized through centralized procedures. In a tissue-engineered product both components (the scaffold and cell product) must be GMP grade and the combination of the two should be also GMP certified. This includes the manufacture and expansion of cells in the scaffold prior implantation, demonstration of conserved mechanical properties and safety/efficacy in adequate animal models. Therefore, it is likely that the first products to become available for clinical experimentation will be those made by clinical grafts containing cells that have been already tested in clinical trials. In term of safety, it should be kept in mind that CHD is increasingly acknowledged to be associated with genetic polymorphisms. Whether alterations in the genetic program that controls the proper formation of the heart may influence stem cell behavior remains unknown and may represent an additional challenge for the progress of the field.

**Figure 5:**
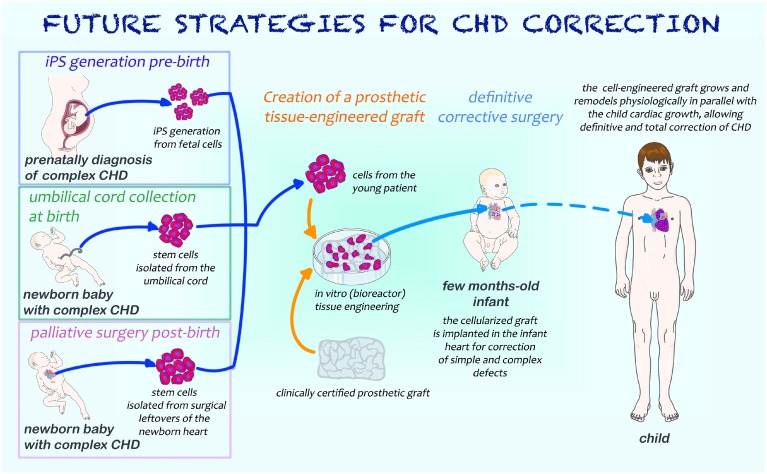
**Cartoon illustrating possible future strategies for the surgical management of newborns with CHD**. If CHD is diagnosed prenatally, foetal cells may be harvested and iPS generated; as an alternative, umbilical cord stem cells can be isolated at the time of birth. When diagnosis of CHD is made after birth or in babies who require a palliative surgical operation soon after birth, stem cells may be isolated from surgical cardiac leftovers. All these types of cells will allow the generation of a tissue-engineered graft endowed with growth and remodeling potential, necessary for the definitive correction of cardiac defects.

Advancements in diagnostic screening allow an early diagnosis of CHD and set the basis for guiding the best reconstructive strategy as illustrated in Figure 5. In the first scenario, cells are obtained prenatally or at birth with minimally invasive procedures, expanded, incorporated in scaffolds ready for use in definitive surgical correction without need of palliative procedures. Induced pluripotent cells could be ideally generated to this purpose. Alternatively, cord cells have a potential for cases that do not require an immediate intervention at birth. In the second scenario, when diagnosis is made post-natally, stem cells from remnants of palliative surgery could be expanded *in vitro* and combined with biomaterials using bioengineering techniques; afterwards, the resulting cellularized graft will be surgically implanted into the diseased heart at the occasion of a second open-chest surgery, helping the surgeon in performing the reconstructive procedures required to manage complex CHD. Looking at the best cell type, the use of stem cells from cardiac source may give the advantage of a physiological commitment to originate all the 3 major cell types of the heart: cardiomyocytes, ECs and VSMCs. Additional cells with complementary activities, namely vascular cells and pericytes could be added in a combinatory approach.

In conclusion, the potential of tissue engineering may provide in the next future definitive solutions for correction of CHD in newborns and children, opening new avenues with immense therapeutic benefits.

## Author contributions

EA: reviewed the literature, drafted the manuscript and prepared the figures; MC: critically revised the manuscript; PM: reviewed the literature, drafted and critically revised the manuscript. All the authors have approved the final submission of the manuscript.

### Conflict of interest statement

The authors declare that the research was conducted in the absence of any commercial or financial relationships that could be construed as a potential conflict of interest.

## Abbreviations

3D: 3-dimensional
BMCs: bone marrow-derived cells
BMMNCs: bone marrow mononuclear cells
BP: bovine pericardium
CHD: congenital heart disease/defect
CPs: cardiac pericytes
CSCs: cardiac stem cells
ECM: extracellular matrix
ECs: endothelial cells
EPCs: endothelial progenitor cells
HF: heart failure
HLHS: hypoplastic left heart syndrome
HPs: heart pericytes
iPS: induced pluripotent stem cells
IVC: inferior vena cava
LV: left ventricle
LVOT: left ventricle outflow tract
MI: myocardial infarction
MMPs: matrix metalloproteinases
MSCs: mesenchymal stem cells
PCL: poly-Caprolactone
PGA: poly-Glicolyc Acid
PLLA: poly-L-Lactic Acid
RA: right atrium
RV: right ventricle
RVEF: right ventricle ejection fraction
RVOT: right ventricle outflow tract
TE: tissue engineering
ToF: Tetralogy of Fallot
UCBMNCs: umbilical cord blood mononuclear cells
VEGF(R): Vascular Endothelial Growth Factor (Receptor)
VICs: valve interstitial cells
VSMCs: Vascular Smooth Muscle Cells.
